# The Impact of Psychosocial Factors on the Work Capacity of Healthcare Workers

**DOI:** 10.1192/j.eurpsy.2025.1576

**Published:** 2025-08-26

**Authors:** I. Kacem, F. Chelly, O. Ajmi, A. Fki, C. Sridi, N. Belhadj Chabbah, H. Ben Hamada, M. Kahloul, W. Naija

**Affiliations:** 1 University of Sousse, Faculty of Medicine Ibn Al Jazzar; 2 Department of Occupational Medicine, Farhat Hached University Hospital; 3 Sahloul University Hospital, Department of Occupational Medicine; 4 Farhat Hached University Hospital, Psychiatry Department; 5 Sahloul University Hospital, Anesthesia and Intensive Care Department, Sousse, Tunisia

## Abstract

**Introduction:**

Healthcare workers, faced with increasingly demanding professional requirements, are particularly exposed to psychosocial risks. These psychosocial factors have direct repercussions on their work capacity, potentially compromising their professional performance and increasing the risk of medical errors.

**Objectives:**

To study the influence of psychosocial factors on the work capacity of healthcare workers.

**Methods:**

This is an analytical cross-sectional study conducted among healthcare staff at Sahloul University Hospital in Sousse over a 3-month period. Our study was based on a questionnaire that included socio-professional characteristics, Karasek’s model, and the Work Ability Index (WAI).

**Results:**

One hundred and thirty-seven staff members were included in this study, with a response rate of 72.4%. The mean age was 48.7 years, with a sex ratio of 0.57. A low work ability (WAI) was reported in 37.1% of cases. A high psychological demand at work was noted in 24.8% of cases. Job strain was reported in 18.1% of cases. Low WAI scores were statistically associated with age (p<10-3), female gender (p<10-3), lack of physical activity (p = 0.03), professional seniority (p<10-3), and high psychological demand at work (p<10-3). No association was found between low decision latitude, low social support, and WAI scores.

**Conclusions:**

The results of this study highlight the significant impact of psychosocial factors on the work capacity of healthcare staff. Preventive measures in healthcare settings to improve working conditions and preserve the mental health of caregivers are essential.

**Disclosure of Interest:**

None Declared

